# Staged Management of Adult Traumatic Atlantoaxial Rotatory Fixation With C2 Superior Facet Fracture: A Case Report

**DOI:** 10.7759/cureus.90981

**Published:** 2025-08-25

**Authors:** Nozomi Akatsu, Hideki Hayashi, Takashi Hanyu, Hiroki Toda

**Affiliations:** 1 Neurosurgery, Medical Research Institute Kitano Hospital, PIIF Tazuke-Kofukai, Osaka, JPN; 2 Neurosurgery, Uji Tokushukai Hospital, Kyoto, JPN; 3 Neurosurgery, Kitano Hospital, Osaka, JPN

**Keywords:** atlanto-axial rotatory fixation, axis fracture, c1-c2 fixation, cervical spine fracture, halo vest

## Abstract

Atlantoaxial rotatory fixation (AARF) is a rare pathological condition predominantly seen in pediatric populations. In adults, particularly when accompanied by C2 facet fractures, it poses unique diagnostic and therapeutic challenges. While conservative treatment may be effective in select cases, the presence of mechanical and/or ligamentous instability often necessitates surgical intervention. We report the case of a 73-year-old male who sustained traumatic AARF with an associated fracture of the right C2 superior articular facet following a bicycle collision. Initial conservative management, including bed rest and analgesia, failed to achieve a reduction. On post-injury day five, closed reduction was performed under general anesthesia and followed by halo vest immobilization. Imaging post-reduction revealed potential instability at the fracture site and a suspected transverse ligament injury. Surgical stabilization via posterior C1-C2 fixation using the Goel-Harms technique was undertaken on day nine. The postoperative course was uneventful, and the patient was discharged on day 52. At the one-year follow-up, alignment was maintained with substantial improvements in pain and neck function. No recurrence of rotational deformity was noted, although complete osseous union of the facet fracture was not achieved. This case illustrates that staged management combining closed reduction and posterior fixation can be effective for complex adult AARF with concurrent facet fractures. Early surgical intervention should be considered in the presence of instability or when conservative measures fail. Individualized treatment planning, informed by anatomical and biomechanical considerations, is critical for optimal outcomes.

## Introduction

AARF is an uncommon pathological condition characterized by painful torticollis and limited cervical motion, typically arising from trauma or upper respiratory infections [1,2]. Prompt and accurate diagnosis is crucial, as delayed recognition may result in chronicity and complicate management [3,4].

AARF is radiologically classified into four types by Fielding and Hawkins based on the degree of rotation and displacement of the atlas relative to the axis [5]. Type I: Pure rotatory fixation without anterior displacement; the transverse ligament is intact. Type II: Rotation with anterior displacement of one lateral mass by 3-5 mm, typically indicating partial transverse ligament insufficiency. Type III: Anterior displacement of both lateral masses exceeding 5 mm, suggesting significant ligament disruption. Type IV: Posterior displacement of the atlas, usually associated with odontoid aplasia.

Conservative treatment, including traction and manual or anesthetized reduction, is generally effective in acute, reducible, and stable cases, particularly in pediatric patients and those with Fielding Type I AARF [1].

When AARF is accompanied by a C2 superior facet fracture, treatment strategies must be meticulously tailored to the specific characteristics of the injury and individual patient factors [6]. In selected acute cases, nonoperative management involving skull or halter traction followed by immobilization with a halo vest or cervical collar can achieve satisfactory realignment while preserving the cervical range of motion [7]. However, chronic, irreducible, or unstable injuries frequently necessitate surgical interventions. Surgical options include posterior C1-C2 fixation using Harms or transarticular techniques, open reduction with or without fusion, and endoscopic anterior (ventral) release in selected cases. Overall, these surgical approaches have demonstrated favorable outcomes, including the restoration of anatomical alignment, achievement of stable fusion, and minimal residual deformity [6].

This study presents a case of AARF associated with a fracture of the C2 superior articular facet, which was effectively managed through a staged treatment approach. The initial intervention involved closed reduction, followed by posterior C1-C2 instrumentation and fusion, culminating in favorable clinical and radiological outcomes. This case underscores the efficacy of integrating conservative and surgical strategies to achieve stable realignment and symptom resolution in patients with complex atlantoaxial injuries.

## Case presentation

**Figure 1:**
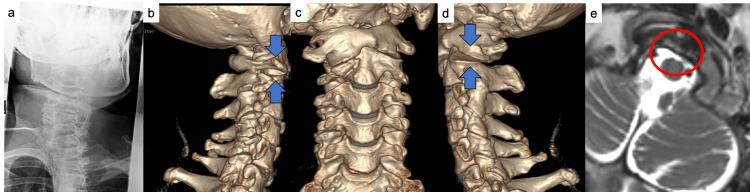
Preoperative findings. (a) The clinical photograph illustrates a fixed rotation of the neck to the left, characteristic of torticollis. (b–d) Three-dimensional CT images reveal a posterior dislocation of the left C1–C2 facet joint and an anterior dislocation of the right C1–C2 facet joint. (e) The axial T2-weighted MRI demonstrates compression of the dural sac and a high-intensity signal posterior to the odontoid process, indicative of transverse ligament injury.

A 73-year-old male was involved in a bicycle-to-bicycle collision, during which he fell to the left and impacted the left side of his head against a utility pole. He was subsequently transported to a nearby hospital via ambulance. Initial imaging revealed cervical spine fractures accompanied by a fixed leftward rotation of the neck. Due to the complexity of the injury, the patient was transferred to our department for specialized treatment. His primary complaint was posterior neck pain, and physical examination indicated a fixed rotation of the neck to the left (Figure 1a), with no spontaneous reduction achievable. There were no neurological deficits, including motor weakness or sensory disturbances in the extremities.

Upon admission, the visual analog scale (VAS) score (0-100 mm) was recorded at 63, while the neck disability index (NDI) (0-100) was at 100. Cervical bone computed tomography (CT) revealed a posterior dislocation at the left C1-C2 facet joint and an anterior dislocation at the right C1-C2 facet joint, indicative of fixed AARF (Figures 1b-1d). Additionally, a fracture dislocation was observed at the right superior articular process of C2, with an atlantodental interval measuring 2.8 mm. Magnetic resonance imaging (MRI) demonstrated compression of the dural sac without intramedullary high-signal alterations. A high T2 signal posterior to the odontoid process suggested an injury to the transverse ligament (Figure 1e). CT angiography indicated mild stenosis of the left vertebral artery at the C2 transverse foramen, consistent with a grade 1 blunt cerebrovascular injury. There was no evidence of posterior circulation infarction, and blood flow was considered preserved; thus, digital subtraction angiography was not conducted. Based on these findings, the diagnosis was confirmed as AARF with a fracture of the right C2 superior articular process.

**Figure 2:**
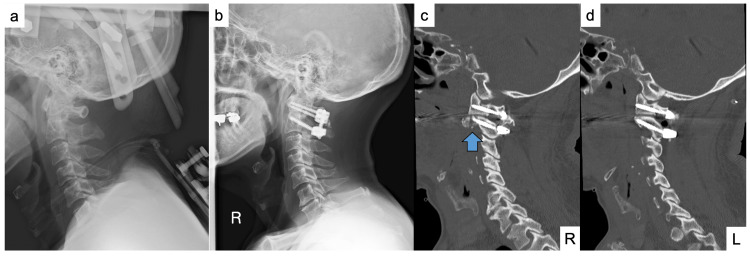
Post-reduction and postoperative imaging. (a) A lateral cervical radiograph following closed reduction confirms the realignment of the C1–C2 facet joints. (b) A postoperative lateral radiograph illustrates proper alignment subsequent to posterior C1–C2 fixation. (c, d) Postoperative sagittal CT images depict the C2 superior articular processes: (c) the right facet appears comminuted with multiple fracture fragments (blue arrow), whereas (d) the left facet exhibits maintained anatomical alignment without overcorrection.

Initial conservative management, consisting of bed rest and analgesia, did not result in spontaneous reduction. On the fifth day post-injury, a closed reduction was conducted under general anesthesia, followed by external immobilization with a halo vest. During the reduction, two distinct "clicks" were audible. Post-reduction imaging confirmed the realignment of the facet joints (Figure 2a), although the right superior articular process was comminuted, and the joint surface was supported at the fracture site. Due to concerns regarding instability, surgical fixation was subsequently performed.

On the ninth day, posterior C1-C2 fixation was executed utilizing the Goel-Harms technique [8]. A navigation reference device was affixed to the C2 spinous process. Bilateral C1 lateral mass screws were positioned with their tips advanced to the anterior arch, and bilateral C2 pedicle screws were placed with their tips advanced to the anterior vertebral body. Tapping was conducted up to 25 mm, and screws were inserted following confirmation of appropriate lengths. A corticocancellous iliac crest bone graft was harvested from the left side. Following decortication, block grafts were positioned between the posterior arches of the C1 and C2 laminae, and bilateral rods were secured using set screws with compression applied to stabilize the grafts. Lateral fluoroscopy and postoperative radiography confirmed adequate alignment without overcorrection (Figures 2b-2d). The postoperative course was uneventful, with no perioperative complications observed.

The halo vest was removed on the 42nd day post-injury, and the patient was discharged on the 52nd day with a modified Rankin Scale score of 0. At the one-year follow-up, alignment was maintained, and pain management was favorable, with a VAS score of 15 and an NDI of 2.5. At this time, cervical bone CT demonstrated that although complete bony union of the C2 superior articular process had not been achieved, healing progression was evident. Screw positioning remained appropriate, and no recurrence of the rotational deformity was identified.

## Discussion

Traumatic AARF, particularly in adults, constitutes an exceedingly rare injury to the upper cervical spine, with its occurrence alongside axial fractures being even more uncommon. These high-energy traumatic incidents, often resulting from motor vehicle collisions or falls, manifest with a wide spectrum of symptoms, ranging from restricted mobility and torticollis to severe neurological deficits, thereby rendering their diagnosis and therapeutic management exceptionally challenging [9-13].

The primary objectives of treatment are to restore a normal, pain-free range of motion, prevent or alleviate neurological impairment, and establish spinal stability. Conservative treatment, typically involving traction (e.g., halo traction) and external immobilization (e.g., halo vest or cervical collar), may be considered an initial therapeutic option in carefully selected cases. This approach is generally indicated when the transverse ligament is intact, no neurological deficits are present, and a successful initial reduction can be achieved and maintained. Several cases of successful conservative management have been reported [7,9,12,14]. However, the non-surgical approach is often insufficient, particularly in cases of significant instability. An inadequate response to such treatment may result in persistent pain, restricted cervical mobility, or recurrent subluxation. Numerous instances have been documented where surgical intervention was either initially undertaken or pursued following the ineffectiveness of conservative treatment (Table 1).

**Table 1:** Summary of reported cases of AARF with associated C2 facet fractures. The table delineates previously documented instances of AARF in conjunction with fractures of the C2 superior articular facet or lateral mass. The first four entries detail cases managed through conservative approaches, whereas the subsequent six entries pertain to surgically treated cases, including the current report. Although conservative treatment yielded favorable outcomes, the follow-up durations were generally brief, and certain cases exhibited residual functional impairments. Conversely, surgical cases consistently demonstrated positive outcomes, notwithstanding residual limitations in rotational motion attributable to instrumentation use. Notably, some cases necessitated surgical intervention following the failure of initial conservative management.

Author	Sex and Age		Mechanism	Other fractures	Management	Time to intervention	Outcome	
Seybold (2003) [9]	Female, 21		Traffic accident	none	Manual reduction → Cervical collar	10 days	Good recovery	
								Spoor (2008) [7]	Male, 43		Bicycle accident	Type II odontoid fracture	Halo traction→Halo vest	Traction same day → reduced after 3 days	Partial relief, Mild right shoulder paresis		
Oh (2010) [14]	Male, 37		Roof tile falling	Type III odontoid fracture	traction＋Halo vest 3 month	Same day	Partial relief, Slight torticollis										
								Bellil (2014) [12]	Female, 56		Traffic accident	none	Manual reduction ＋Halo vest 3month	3 days	Good recovery		
Kevin & Moore (1995) [15]	Male, 65		Automobile accident	C2 right transverse process	Gardner-Wells Tong and traction →C1-C2 posterir wiring and fusion	Same day	Good recovery										
Kim (2017) [11]	Male, 34		Fall from stairs	none	Reduction＋C1-C2 pedicle screw＋Brooks	Several days	Good recovery		
Alhasani (2022) [13]	Male, 41		Traffic accident	none	Harms （C1-C2-C3 fixation）	Next day	Partial relief, Neck pain during exercise		
								Eghbal (2024) [10]	Male, 27		Traffic accident	none	C1-C2 fixation	Surgery after conservartive treatment	Good recovery		
Peyriere (2017) five cases [6]	Male 2 Female 3	27-82	Falls, hang glider, epileptic seizure, etc.	not specified	Harms or Magerl	3： within 1 week,	Good recovery										
						1：15 days			
						1： 3 months			
Present case	Male, 73		Bicycle accident	none	Reduction under general anesthesia, Halo vest, C1-C2 fixation (Harms)	Halo vest after 5 days, fixation after 9 days	mRS 0		

Moore and Frank reported a case of traumatic AARF in a 65-year-old patient who sustained fractures of the C2 lateral mass and transverse process following a motor vehicle accident. A closed reduction was successfully achieved within 24 hours through cervical extension. Subsequently, at the patient's request, posterior C1-C2 fusion was performed due to his refusal of halo fixation [15].

Kim et al. reported a case of type II post-traumatic AARF in an adult, where closed reduction using skull traction and gentle manipulation was ineffective. Surgical intervention involving open reduction and C1-C2 transpedicular screw fixation, in conjunction with posterior wiring, resulted in the complete resolution of torticollis and the restoration of normal alignment. Postoperative radiographic evidence confirmed fusion, and no neurological deficits were observed [11].

In a similar case, Alhasani et al. documented a 41-year-old male patient presenting with C1-C2 rotatory subluxation and a locked facet subsequent to a traffic accident. Following an unsuccessful attempt at closed reduction, a posterior C1-C2 fusion was executed using the Harms technique, which successfully restored cervical mobility and ameliorated torticollis. The authors recommend surgical intervention in instances where closed reduction is ineffective due to mechanical locking or in Type II and more severe forms of AARF with transverse ligament insufficiency [13].

Eghbal et al. conducted a case series involving five adult patients diagnosed with traumatic atlantoaxial rotatory subluxation. Initially, all patients received conservative treatment, which included skull traction and orthotic immobilization. However, due to insufficient clinical improvement, surgical intervention became necessary. Posterior fixation resulted in significant functional enhancement and symptom resolution in all patients, underscoring the limitations of conservative approaches in adult cases [10].

Peyriere et al. documented five adult instances of post-traumatic AARF accompanied by C2 articular facet fractures. Posterior screw fixation, specifically the Harms technique in four cases, resulted in favorable clinical and radiological outcomes. Notably, they proposed that permanent fusion might not be requisite in these cases due to the absence of ligamentous injury. In such circumstances, the removal of implants following bone healing could be considered to maintain upper cervical mobility [6].

While favorable outcomes with conservative management strategies, such as traction or halo vest immobilization, have been documented in the literature, these reports often involve short-term follow-ups. The potential for delayed cervical instability, along with associated symptoms or functional deterioration, may become apparent during an extended observation period. Consequently, while nonoperative management remains an option for selected cases, adult patients with AARF complicated by facet interlocking, osseous fractures, or suspected ligamentous injury often require surgical stabilization.

**Figure 3:**
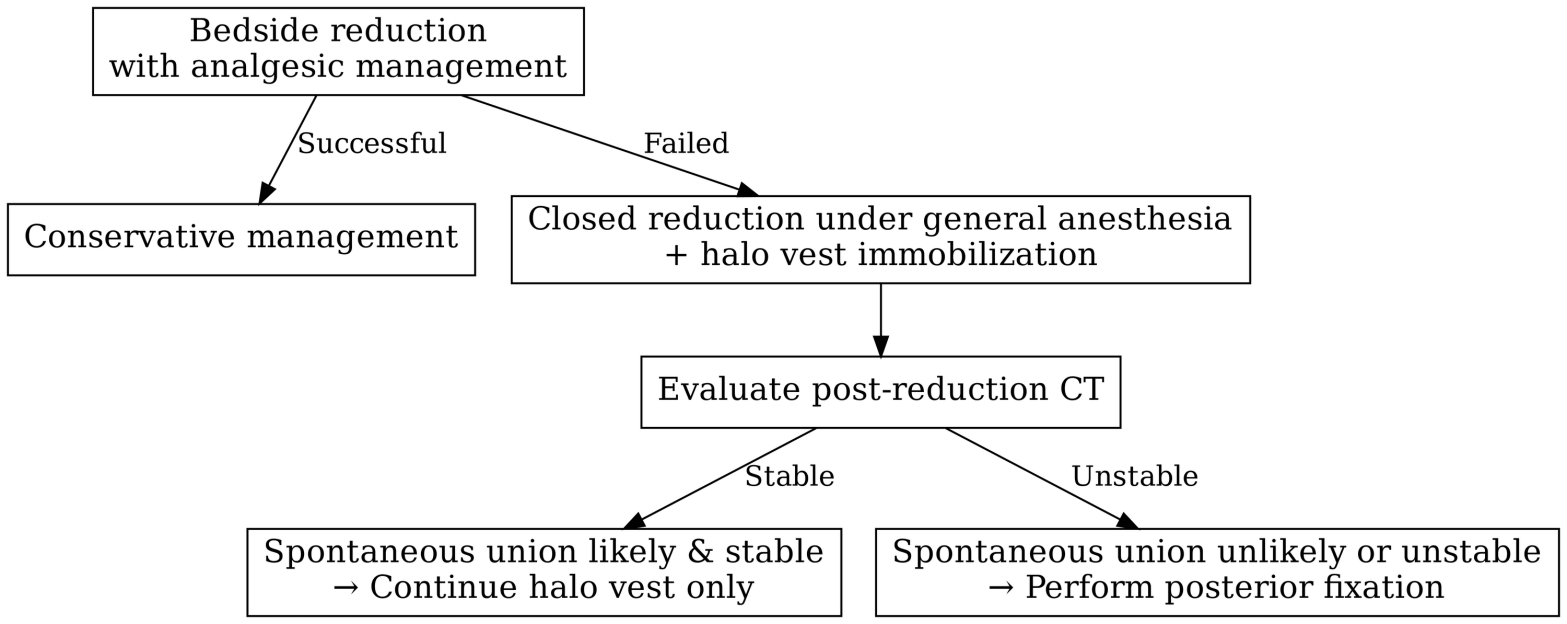
Staged treatment strategy for the present case. The figure was created by the authors.

In light of these considerations, our initial strategy involved conservative management using analgesics to facilitate closed manual reductions (Figure 3). However, this approach is impeded by fixed rotational deformity, necessitating closed reduction under general anesthesia, followed by halo vest immobilization. Post-reduction CT revealed a comminuted fracture of the right C2 superior articular process, raising concerns regarding mechanical instability. Additionally, the initial MRI demonstrated a high T2 signal intensity posterior to the odontoid process, indicative of transverse ligament disruption. These findings corroborate the presence of both osseous and ligamentous instability. We assessed the likelihood of spontaneous bone union based on fracture morphology reduction. If the CT indicated sufficient cortical apposition, we continued halo vest immobilization; however, in this instance, the fragmented nature of the articular process rendered union unlikely, and persistent instability at the C1-C2 joint was anticipated. Given the risk of nonunion and the potential for serious complications, including medullary compression or vertebral artery injury, we proceeded with posterior C1-C2 fixation. The patient’s postoperative course was favorable, with substantial improvement in neck pain and no neurological deficits, although mild rotational restriction persisted owing to instrumentation.

Although early surgical intervention was a valid option, we carefully considered its disadvantages. Posterior fusion can result in a reduced cervical range of motion and carries risks such as implant-related infection and vertebral artery injury. Therefore, our institutional protocol favors an initial attempt at reduction and external immobilization if alignment is achievable and bone healing is likely.

In scenarios where fixation is not performed despite poor union potential, prolonged external immobilization may be necessary, negatively impacting the patient’s quality of life and increasing the risk of disuse syndrome. In our case, a complete union of the right superior articular process was not observed. Without surgical intervention, halo vest removal would have been challenging or would have resulted in secondary complications.

This case report is subject to several limitations. First, it pertains to a single patient, which inherently limits the generalizability and external validity of the findings. Broader conclusions regarding the efficacy and reproducibility of the treatment strategy cannot be drawn without the further accumulation of similar cases and extended longitudinal follow-up. Second, although the patient exhibited favorable clinical and radiographic outcomes at the one-year postoperative evaluation, the long-term integrity of osseous fusion, potential for delayed cervical instability, and the risk of other late-onset complications remain undetermined. Third, while initial nonoperative management was attempted, a comparative assessment of alternative conservative modalities, such as sustained traction or different forms of external immobilization, was not feasible. Notably, continuous traction was considered but could not be implemented due to equipment limitations at our institution. Further studies are required to establish optimal management strategies, particularly in resource-constrained settings.

## Conclusions

This case underscores the importance of considering early surgical intervention in adult traumatic AARF with an associated C2 facet fracture, particularly when instability is suspected and conservative treatment proves ineffective. Surgical stabilization is associated with favorable clinical and radiological outcomes. Accurate diagnosis, meticulous planning, and individualized management are essential for ensuring long-term stability and functional recovery.
